# Supplementary material for: Patched 1 reduces the accessibility of cholesterol in the outer leaflet of membranes
Source: eLife. 2021 Oct 26;10:e70504. doi: 10.7554/eLife.70504 (PMC8654371; doi:10.7554/eLife.70504)
Supplement: Figure 3—source data 1. — As denoted on the left immunoblot PTCH1 (ptc) samples were run in duplicate. [file elife-70504-fig3-data1.pdf]

Figure 2- Source Data 1

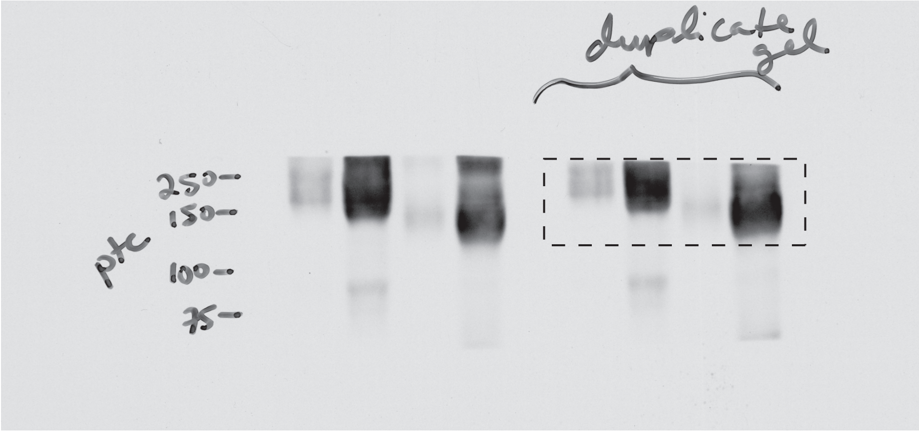

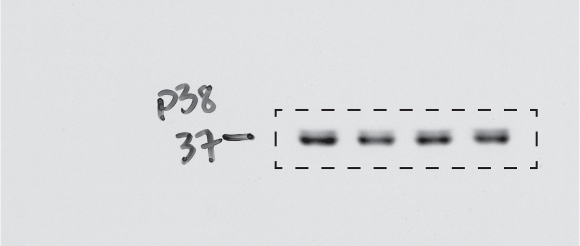

Figure 2--Source Data 1. Uncropped scans from immunoblots shown in Figure 2B. Dotted lines denote the cropped region of the immunoblot that is shown Figure 2B. As denoted on the left immunoblot PTCH1 (ptc) samples were run in duplicate.
