# Supplementary material for: Patched 1 reduces the accessibility of cholesterol in the outer leaflet of membranes
Source: eLife. 2021 Oct 26;10:e70504. doi: 10.7554/eLife.70504 (PMC8654371; doi:10.7554/eLife.70504)

PTCH1, PFOD4H and P38 blot showing cropping used in Figure 3- figure supplement 1 in dashed boxes. Two exposures were used in the figure with the darker exposure (right) used to shown PFOD4H. PFOD4H was detected by blotting for the TdTomato fusion protein. TdTomato-PFOD4H is expected to run at ~65 kDa.

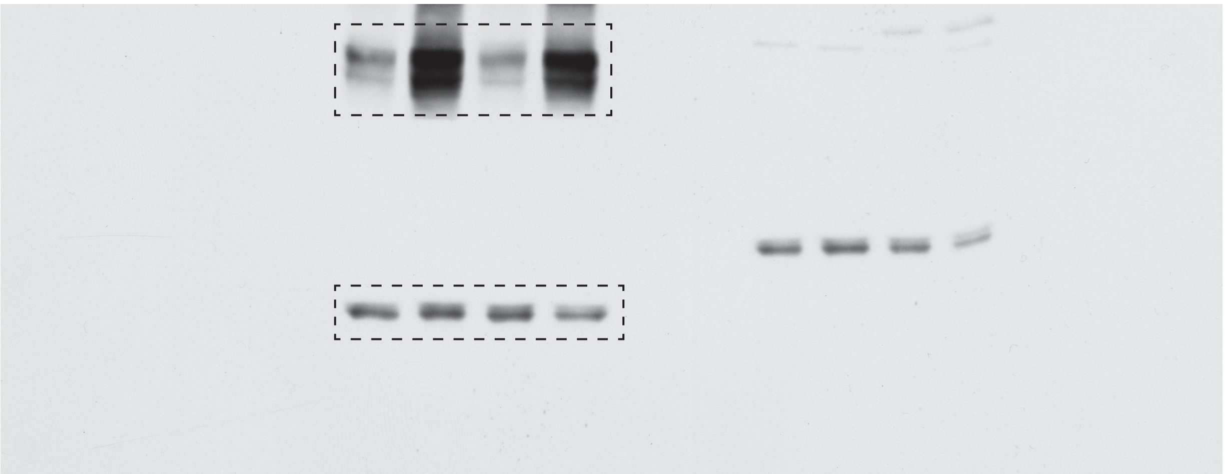

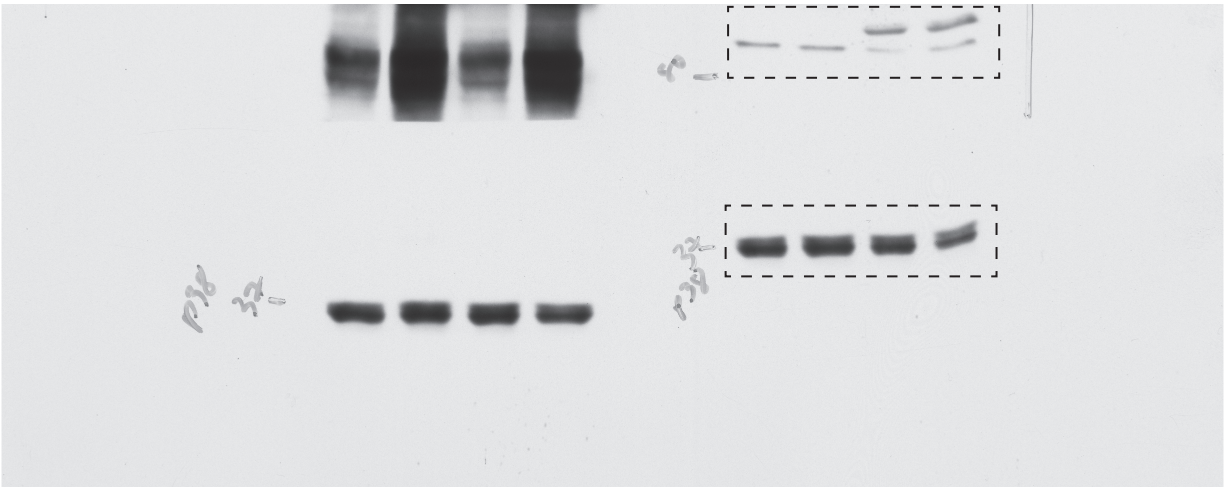

Supplement: Figure 3—figure supplement 1—source data 1. — Dotted lines mark the cropped region of the immunoblot that was used to generate panel Figure 3—figure supplement 1A. [file elife-70504-fig3-figsupp1-data1.zip › PFOD4 uncropped blots_FigureS3_1A.pdf]
