# Supplementary figures and images for: Patched 1 reduces the accessibility of cholesterol in the outer leaflet of membranes
Source: eLife. 2021 Oct 26;10:e70504. doi: 10.7554/eLife.70504 (PMC8654371; doi:10.7554/eLife.70504)

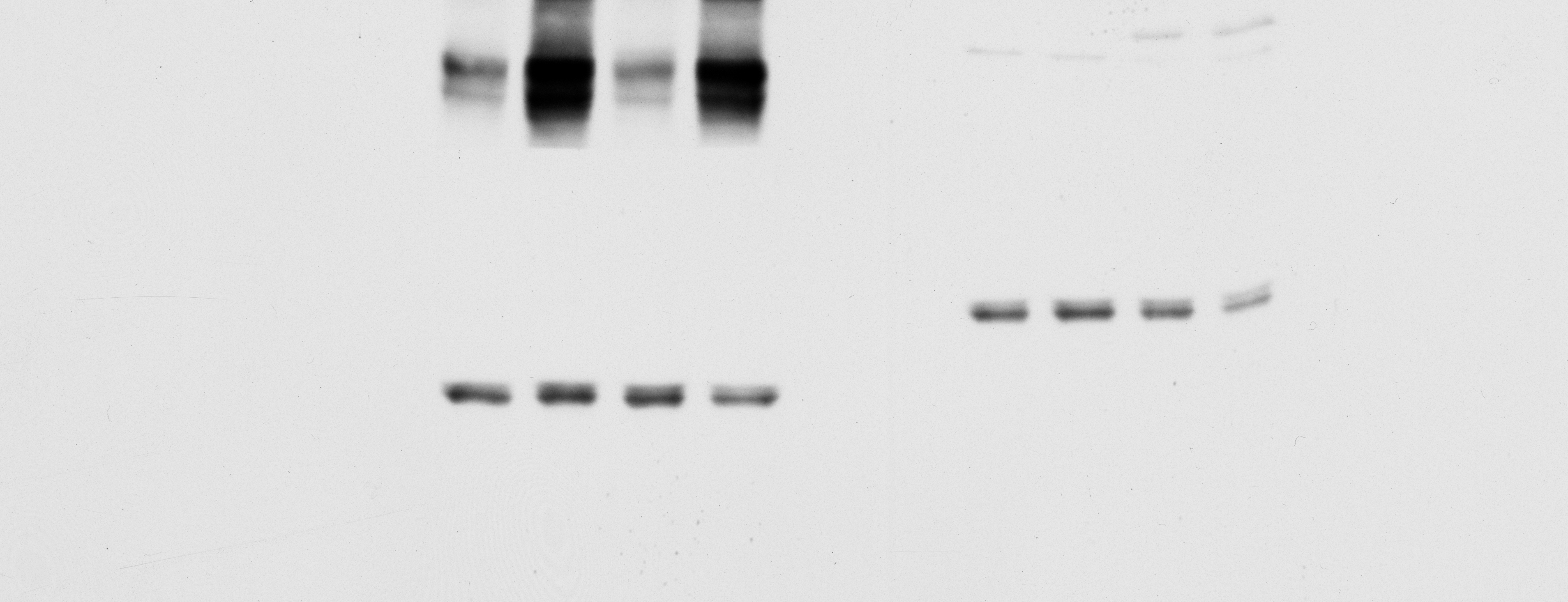

Supplement: Figure 3—figure supplement 1—source data 1. — Dotted lines mark the cropped region of the immunoblot that was used to generate panel Figure 3—figure supplement 1A. [file elife-70504-fig3-figsupp1-data1.zip › 09_15_21__003_FigureS3_1.tiff]

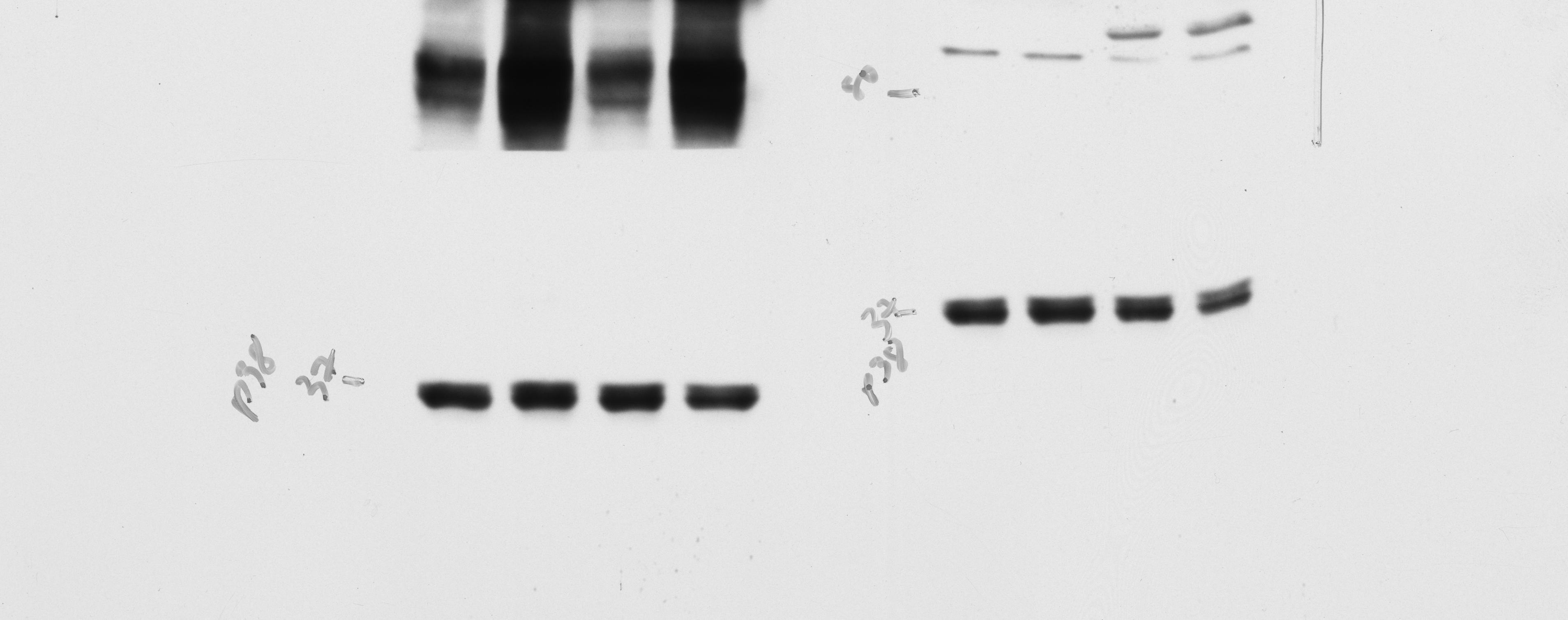

Supplement: Figure 3—figure supplement 1—source data 1. — Dotted lines mark the cropped region of the immunoblot that was used to generate panel Figure 3—figure supplement 1A. [file elife-70504-fig3-figsupp1-data1.zip › 09_15_21__003_FigureS3.tiff]
