# Supplementary material for: Patched 1 reduces the accessibility of cholesterol in the outer leaflet of membranes
Source: eLife. 2021 Oct 26;10:e70504. doi: 10.7554/eLife.70504 (PMC8654371; doi:10.7554/eLife.70504)
Supplement: Figure 5—source data 1. — Dotted lines mark the cropped region of the immunoblot that was used to generate panel Figure 5B. [file elife-70504-fig5-data1.pdf]

Figure 4- Source Data 1

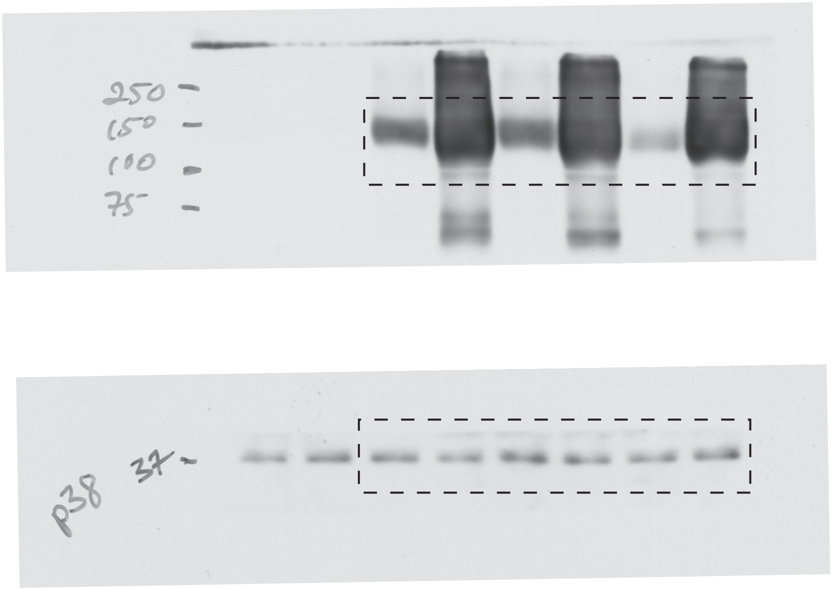

Figure 4--Source Data 1. Uncropped scans from immunoblots shown in Figure 4B. Dotted lines denote the cropped region of the immunoblot that is shown Figure 4B.
